# Untapping root system architecture for crop improvement

**DOI:** 10.1093/jxb/erw262

**Published:** 2016-08-03

**Authors:** Frank Hochholdinger

**Affiliations:** Institute for Crop Science and Resource Conservation (INRES), Faculty of Agriculture, University of Bonn, 53177 Bonn, Germany

**Keywords:** Crown root number, drought, maize, rooting depth, water deficit

**Drought leads to greater yield loss in crops than any other abiotic stress. Roots form a plant’s first line of defense against water deficit, and in this issue Gao and Lynch (pages 4533–4546) report that fewer but longer crown roots facilitate water acquisition from subsoil and thus improve drought tolerance in maize.**

Roots are the first organ to sense water deficit, and at the same time they extract virtually all those mineral nutrients from soil that are consumed by humans. Soil resource acquisition under challenging conditions such as drought is a primary limitation on plant productivity, resulting in low yields and thus food insecurity in the poorest countries. Indeed, in overall global agriculture, drought accounts for more loss in plant productivity than any other abiotic factor. Thus the development of crop cultivars with enhanced traits for soil resource acquisition is a key goal for feeding the growing world population (Lynch, 2007). However, so far the potential of root traits for crop improvement remains largely unexploited, largely because of the limited accessibility of roots in their belowground soil habitat.

## Maize roots and resource acquisition

Agronomically important cereals such as maize have a particularly sophisticated root architecture composed of several root types formed at different stages of development (see Hochholdinger and Tuberosa, 2009). The 3D structure of root systems which ensures optimal capture of soil resources is determined by intrinsic regulators and external triggers fluctuating in space and time. Their developmental flexibility provides a mode of action allowing rapid architectural adjustment as environmental conditions change. In maize, the shoot-borne root system emerging from consecutive below- and aboveground shoot nodes is of particular importance because it makes up the backbone of the adult root stock (see Hochholdinger *et al.*, 2004) (Box 1). In recent years, complementary approaches have focused on the genetic, phenomic, anatomical and physiological characterization of shoot-borne root formation in maize and the role of these roots in resource acquisition under challenging conditions.

**Box 1. The shoot-borne root system of maize**

Field-excavated root stock of a 10-week-old maize plant at anthesis. At this developmental stage, postembryonically formed shoot-borne roots form the backbone of the maize root system, with the number genotype-dependent and ranging from as few as five to >70. Shoot-borne roots formed in soil are designated crown roots, while shoot-borne roots initiated above soil level are termed brace roots. Photo courtesy of Jonathan Lynch.

**Figure:**
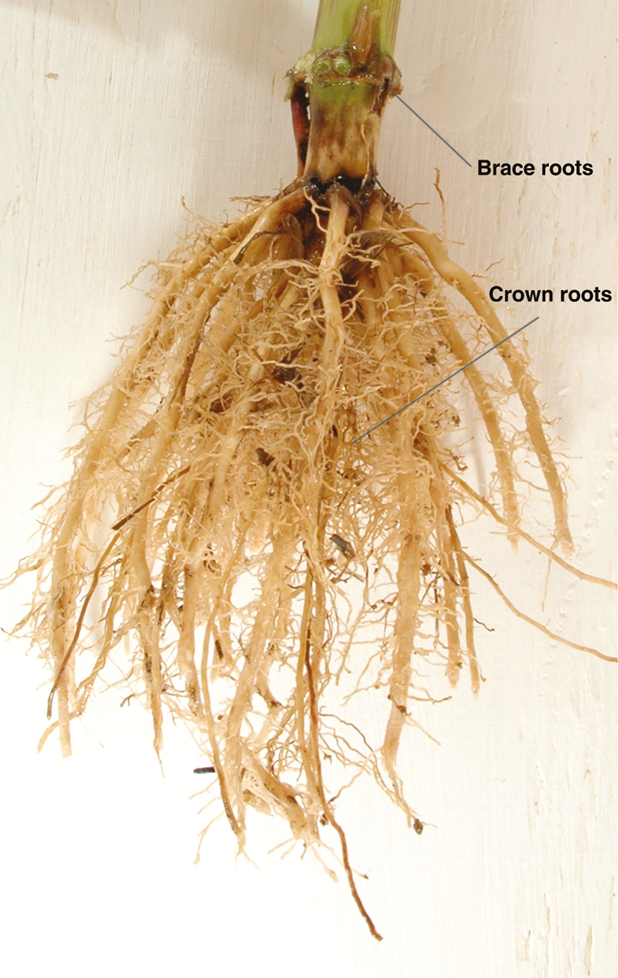

Genetic experiments in maize have demonstrated that *rtcs* (*rootless concerning crown and seminal roots*) is a key checkpoint of shoot-borne root initiation (Hetz *et al.*, 1996), while its paralog *rtcl* (*rtcs-like*) functions in shoot-borne root elongation (Xu *et al.*, 2015). Both genes encode LOB-domain proteins, which belong to a wider family whose members act as central regulators of auxin signal transduction (Taramino *et al.*, 2007). While these genes are master regulators for the initiation and elongation of individual shoot-borne roots, the number of these roots is highly variable and ranges from five to >70 (see references in Gao and Lynch, 2016) between different genotypes, suggesting that the overall total is controlled by multiple genes.

Phenomic, anatomical and physiological experiments have been conducted to define hypothetical maize ideotypes optimized for resource acquisition. An ideotype termed ‘steep, cheap, and deep’ was postulated to perform best under limited water availability in drying soil (reviewed in Lynch, 2013). ‘Steep’ and ‘deep’ refer to morphological adjustments by steep root-growth angles and extended elongation of shoot-borne roots to access water in deeper soil layers; ‘cheap’ refers to cellular adjustments to reduce the metabolic costs of soil exploration, such as a decreased number of cortical cell files, increased cortical cell size and the formation of root cortical aerenchyma (Lynch, 2015).

## Ideotypes for superior performance during drought

In this issue of *Journal of Experimental Botany*, Gao and Lynch (2016) elegantly confirm the desirable ‘steep, cheap, and deep’ ideotype for shoot-borne roots of maize experimentally. The authors demonstrate that maize genotypes with an intermediate number of crown roots grow deeper and therefore have an improved water acquisition capacity under drought conditions, and that this holds true not only in the greenhouse but also in field experiments. They also show that maize plants exposed to prolonged periods of drought stress form a smaller number of shoot-borne roots than plants grown under control conditions. This further suggests that it is not just those genotypes with fewer, longer shoot-borne roots that are superior under drought conditions, but that a more reduced number is also a positive adaptation.

An intermediate number of crown roots is essential for the superior performance of plants. If the number of these roots is too low, plants become susceptible to lodging; if the number is too large, competition for soil and metabolic resources becomes uneconomic. Consequently, under field conditions, genotypes with fewer but deeper crown roots, which have an improved capacity of water capture, display greater shoot biomass and a significantly increased yield (57%) compared with genotypes with higher crown root numbers. Similarly, genetic experiments in rice have demonstrated that the *DEEPER ROOTING 1* (*DRO1*) gene, which underlies a quantitative trait locus (QTL) controlling growth angle, regulates cell elongation at the root tip and downward bending of the root in response to gravitropism. Therefore, higher expression of *DRO1* results in roots growing in a more-downward direction, thus avoiding drought, and an increased yield in these plants (Uga *et al.*, 2013).

A remarkable aspect of the work of Gao and Lynch (2016) is their finding that crown root number and rooting depth correlate significantly. This is of particular interest because the specification of crown root number and their subsequent elongation are different developmental processes. Nevertheless, both processes are beneficial under drought conditions, reducing the metabolic costs of soil exploration. Similarly, the coordinated regulation of distinct developmental aspects of maize root development has also been observed in several monogenic mutants. For instance, the previously mentioned maize *rtcs* gene not only controls the initiation of postembryonic crown roots, but also the initiation of embryonic seminal roots (Hetz *et al.*, 1996). Moreover, the *rootless with undetectable meristem* (*rum1*) mutant is impaired in postembryonic lateral root formation in the primary root, but also in embryonic seminal root initiation (Woll *et al.*, 2005). It could be hypothesized that the coordinated control of several root types across embryonic and postembryonic development by these genes might also be related to the efficient management of metabolic costs, because these functions have developed over long periods of evolutionary time.

## The least understood organs

Despite their major importance for the adaptation of plants to natural or agricultural environments, roots are the least understood of all plant organs. However, scientists from different disciplines, including molecular genetics, physiology and phenomics, are currently assembling a growing body of data to overcome this knowledge gap. An interdisciplinary exchange of ideas and concepts among these scientists would be a major leap forward in understanding the principles underlying the dynamic adaptive responses controlling root structure and function in cereals. The vision of such an exchange would be to obtain a full understanding of how exogenous signals are translated into cellular responses, and how this knowledge can be implemented into future root-based increases in plant productivity.

A first step towards this goal will be a detailed understanding of the physiological, morphological and cellular responses of plants to external stress factors such as drought. Cutting-edge phenotyping and sensor technologies will allow us to monitor very subtle differences during development, paving the way for geneticists and breeders to identify underlying genes or quantitative trait loci (QTLs) (Burton *et al.*, 2014). Moreover, allelic variation in genes with known function in root development can be exploited for the selection of maize lines with improved root architecture (e.g. Kumar *et al.*, 2014). Another resource for candidate genes involved in shoot-borne root architecture of cereals such as maize might come from transcriptome analyses (e.g. Muthreich *et al.*, 2013). Knowing the underlying genes controlling the root stock response to drought will allow a full exploitation of the natural genetic variation of these traits or the introduction of beneficial alleles into elite cultivars using novel approaches such as CRISPR/Cas9 (Liu *et al.*, 2016). For maize in particular, it has to be kept in mind that commercial production is exclusively based on hybrids, because they perform better than their homozygote parents. Moreover, several root traits of maize display heterosis early in development (Paschold *et al.*, 2010). Therefore, the behavior of these traits and genes will have to be tested in highly heterozygous heterotic hybrids.

## References

[CIT0001] Burton AL Johnson JM Foerster JM Hirsch CN Buell CR Kaeppler SM Brown KM Lynch JP. 2014 QTL mapping and phenotypic variation for root architectural traits in maize (*Zea mays* L.). Theoretical and Applied Genetics 128, 93–106. 25230896 10.1007/s00122-014-2353-4

[CIT0001b] Gao Y Lynch JP 2016 Reduced crown root number improves water acquisition under water deficit stress in maize (Zea mays L.). Journal of Experimental Botany 67, 4533–4546. 10.1093/jxb/erw243 PMC4973737 27401910

[CIT0002] Hetz W Hochholdinger F Schwall M Feix G. 1996 Isolation and characterization of *rtcs*, a maize mutant deficient in the formation of nodal roots. The Plant Journal 10, 845–857.

[CIT0003] Hochholdinger F Park WJ Sauer M Woll K. 2004 From weeds to crops: genetic analysis of root development in cereals. Trends in Plant Science 9, 42–48. 14729218 10.1016/j.tplants.2003.11.003

[CIT0004] Hochholdinger F Tuberosa R. 2009 Genetic and genomic dissection of maize root development and architecture. Current Opinion in Plant Biology 12, 172–177. 19157956 10.1016/j.pbi.2008.12.002

[CIT0005] Kumar B Abdel-Ghani AH Pace J Reyes-Matamoros J Hochholdinger F Lübberstedt T. 2014 Association analysis of single nucleotide polymorphisms in candidate genes with root traits in maize (*Zea mays* L.) seedlings. Plant Science 224, 9–19. 24908501 10.1016/j.plantsci.2014.03.019

[CIT0006] Liu D Hu R Palla KJ Tuskan GA Yang X. 2016 Advances and perspectives on the use of CRISPR/Cas9 systems in plant genomics research. Current Opinion in Plant Biology 30, 70–77. 26896588 10.1016/j.pbi.2016.01.007

[CIT0007] Lynch JP. 2007 Roots of the second green revolution. Australian Journal of Botany 55, 493–512.

[CIT0008] Lynch JP. 2013 Steep, cheap and deep: an ideotype to optimize water and N acquisition by maize root systems. Annals of Botany 112, 347–357. 23328767 10.1093/aob/mcs293 PMC3698384

[CIT0009] Lynch JP. 2015 Root phenes that reduce the metabolic costs of soil exploration: Opportunities for 21st century agriculture. Plant Cell & Environment 38, 1775–1784. 10.1111/pce.12451 25255708

[CIT0010] Muthreich N Majer C Beatty M Paschold A Schützenmeister A Fu Y Malik WA Schnable PS Piepho H-P Sakai H. 2013 Comparative transcriptome profiling of maize coleoptilar nodes during shoot-borne root initiation. Plant Physiology 163, 419–430. 23843603 10.1104/pp.113.221481 PMC3762660

[CIT0011] Paschold A Marcon C Hoecker N Hochholdinger F. 2010 Molecular dissection of heterosis manifestation during early maize root development. Theoretical and Applied Genetics 120, 383–388. 19526205 10.1007/s00122-009-1082-6

[CIT0012] Taramino G Sauer M Stauffer Jr JL Multani D Niu X Sakai H Hochholdinger F. 2007 The maize (*Zea mays* L.) *RTCS* gene encodes a LOB domain protein that is a key regulator of embryonic seminal and post-embryonic shoot-borne root initiation. The Plant Journal 50, 649–659. 17425722 10.1111/j.1365-313X.2007.03075.x

[CIT0013] Uga Y Sugimoto K Ogawa S 2013 Control of root system architecture by DEEPER ROOTING 1 increases rice yield under drought conditions. Nature Genetics 45, 1097–1102. 23913002 10.1038/ng.2725

[CIT0014] Woll K Borsuk LA Stransky H Nettleton D Schnable PS Hochholdinger F. 2005 Isolation, characterization, and pericycle-specific transcriptome analyses of the novel maize lateral and seminal root initiation mutant *rum1* . Plant Physiology 139, 1255–1267. 16215225 10.1104/pp.105.067330 PMC1283763

[CIT0015] Xu C Tai H Saleem M Ludwig Y Majer C Berendzen KW Nagel KA Wojciechowski T Meeley RB Taramino G. 2015 Cooperative action of the paralogous maize lateral organ boundaries (LOB) domain proteins RTCS and RTCL in shoot borne root formation. New Phytologist 207, 1123–1133. 25902765 10.1111/nph.13420

